# Humans Can Adopt Optimal Discounting Strategy under Real-Time Constraints

**DOI:** 10.1371/journal.pcbi.0020152

**Published:** 2006-11-10

**Authors:** N Schweighofer, K Shishida, C. E Han, Y Okamoto, S. C Tanaka, S Yamawaki, K Doya

**Affiliations:** 1 Biokinesiology and Physical Therapy, University of Southern California, Los Angeles, United States of America; 2 Department of Psychiatry and Neurosciences, Hiroshima University, Hiroshima, Japan; 3 Computer Science, University of Southern California, Los Angeles, United States of America; 4 Bioinformatics and Genomics, Nara Institute of Science and Technology, Nara, Japan; 5 Department of Computational Neurobiology, Advanced Telecommunications Research Institute International Computational Neuroscience Labs, Kyoto, Japan; 6 Initial Research Project, Okinawa Institute of Science and Technology, Okinawa, Japan; University College London, United Kingdom

## Abstract

Critical to our many daily choices between larger delayed rewards, and smaller more immediate rewards, are the shape and the steepness of the function that discounts rewards with time. Although research in artificial intelligence favors exponential discounting in uncertain environments, studies with humans and animals have consistently shown hyperbolic discounting. We investigated how humans perform in a reward decision task with temporal constraints, in which each choice affects the time remaining for later trials, and in which the delays vary at each trial. We demonstrated that most of our subjects adopted exponential discounting in this experiment. Further, we confirmed analytically that exponential discounting, with a decay rate comparable to that used by our subjects, maximized the total reward gain in our task. Our results suggest that the particular shape and steepness of temporal discounting is determined by the task that the subject is facing, and question the notion of hyperbolic reward discounting as a universal principle.

## Synopsis

When we make a choice between two options, we compare the values of their outcomes and select the option with a larger value. However, what if one option leads to a larger delayed reward and the other leads to a smaller more immediate reward? Naturally, we assign a larger value for a larger reward, but it is “discounted” if the reward is to be delivered later. Thus, the value is a monotonically decreasing function of the delays. Previous behavioral studies have repeatedly demonstrated that humans and animals discount delayed rewards hyperbolically. This has practical importance, as hyperbolic discounting can sometimes lead to “irrational” preference reversal: for instance, an individual may prefer two apples in 51 days to one apple in 50 days, but if the days come closer, he prefers one apple today to two apples tomorrow. On the contrary, exponential discounting is always “rational,” as it predicts constant preference. Here, in a new task that mimics animal foraging, and that uses delayed monetary rewards, Schweighofer and colleagues showed that humans can also discount reward exponentially. Furthermore, it is remarkable that by adopting exponential discounting, their subjects maximized their total gain. Thus, depending on the task at hand, the authors' study suggests that humans can flexibly choose the type of reward discounting, and can exhibit rational behavior that maximizes long-term gains.

## Introduction

In the limited amount of time available before nighttime, winter, or retirement, we need to make a large number of choices to maximize our total reward gain. In particular, when choosing between a larger, but delayed, reward, and a smaller, but more immediate reward, we compare the values associated with each reward, and choose the reward associated with the larger value [1]. Critical to these choices are the *shape* and the *steepness* of the reward values, which monotonically decrease as a function of the delay: the rewards are said to be discounted as a function of the delays (Figure 1A).

**Figure 1:**
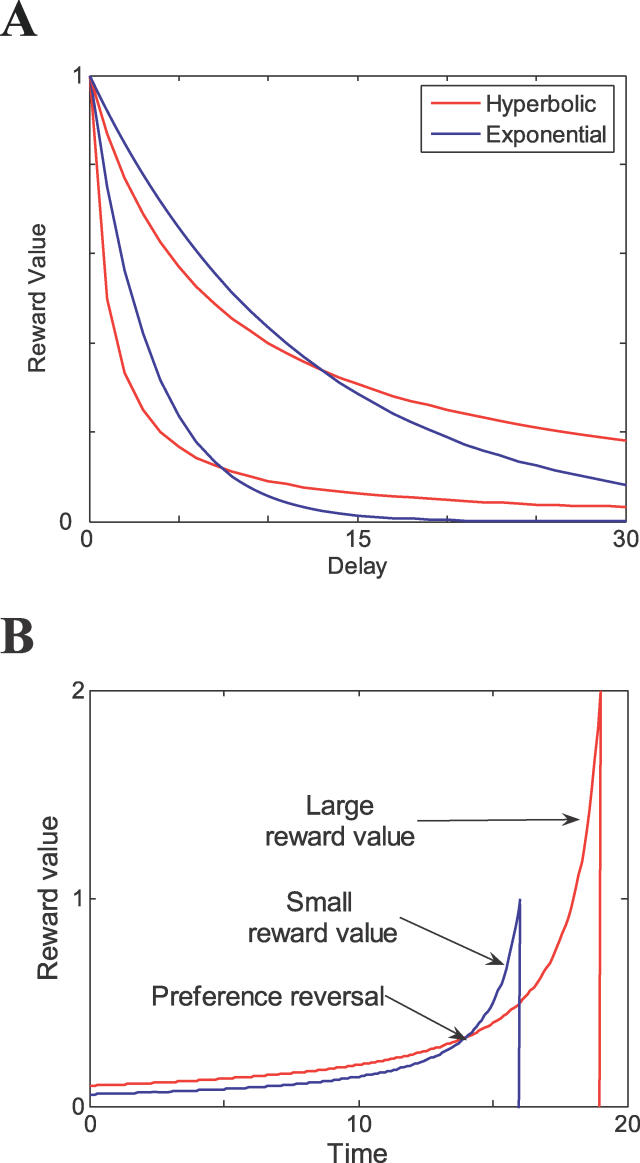
Hyperbolic and Exponential Reward Discounting Models (A) Hyperbolic versus exponential reward discounting models as a function of the delay to the reward for two different sets of steepness parameters. The hyperbolic model has an initial steep decay followed by a flatter “tail”; thus, delayed rewards are less discounted with hyperbolic models than with exponential models. (B) Preference reversal, which is commonly observed in humans and animals, is predicted by the hyperbolic model and is due to a decrease in the decay rate as the delay increases. Initially (at time 0), the large reward has a higher value than the small reward, and is therefore preferred. As the small reward draws near, the preference shifts to the small reward. The exponential model, which has a constant decay rate, does not predict preference reversal.

Two main classes of models that characterize the *shape* of reward discounting have been proposed: exponential [2–4] and hyperbolic [5–13]. Although researchers in artificial intelligence favor exponential discounting in uncertain environments, e.g., [4,14,15], all behavioral studies that have directly compared the two types of discounting in animals or humans have concluded that hyperbolic discounting better fits delayed reward choice data than does exponential discounting, e.g., [6–8,16–18].

In exponential discounting, the reward value *V* is given by:

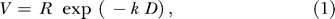
where *R* is the reward magnitude, *D* the delay, and *k* ≥ 0 the decay rate. This equation is equivalently given by:

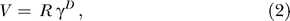
where *γ* is the discount factor (0 ≤ *γ* < 1), and *γ* = exp(−*k*); we note here that a large decay rate corresponds to a small discount factor and vice versa. Because of constant decay rate, exponential discounting is “rational,” as it predicts constant preference.

Typical human studies are questionnaire-based: subjects are asked to make a number of choices between small immediate rewards and larger rewards weeks, months, or years in the future, after thinking about the consequences of each alternative [19] (but see [20]). In these studies, the hyperbolic discounted reward value is given by:

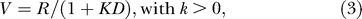



In animal studies, animals are trained to make repeated reward choices, and experience both delays (on the order of a few dozen seconds) and rewards. Assuming a constant inter-trial interval *(ITI),* if the animal consistently makes a choice that gives the same reward *R* after the same delay *D,* the average reward rate is the hyperbolic function of the delay [21]:

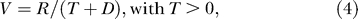
where *T* is the sum of all times except the delay in each trial (*T* is often equal to the *ITI*), and *V* the reward value. Because of the decreasing decay rate as a function of delay [22], hyperbolic discounting has been termed “irrational,” as it predicts preference reversal and impulsive choice (Figure 1B). For instance, an individual may prefer one apple today to two apples tomorrow, but at the same time prefer two apples in 51 days to one apple in 50 days [23]. Hyperbolic discounting is often presented as a struggle between oneself and one's alter ego in the future, or similarly, between a myopic doer and a farsighted planner—see [23,24].

In what situations is it theoretically advantageous to make delayed reward choices based on exponential or hyperbolic discounting? Exponential discounting maximizes total gain in situations of constant probability of reward loss per unit time, and exact estimate of the time of the future reward delivery—see [21,25]. Because hyperbolic discounted value, as given by Equation 4, is the reward rate, it maximizes the total gain in situations of constant delays at each trial (with no reward loss and with an exact estimate of the time of future reward delivery).

But does hyperbolic discounting maximize the total gain in foraging-like situations, that is, in situations of repeated forced choices with varying delays to the rewards, constant *ITI,* and limited total time? In these situations, the hyperbolic discounting model maximizes the instantaneous reward rate. But, as the trials are not independent from each other, hyperbolic discounting may not maximize the average reward rate, and thus the total gain. For instance, in a relatively unfavorable trial with long delays to both rewards, although hyperbolic discounting may favor the large reward, pursuing the small more immediate reward may result in a smaller overall decrease of the average reward rate. By choosing the small but less-delayed reward, the subject can quickly move to the next (hopefully) more favorable trials. Thus, we hypothesize that, in these situations, a discounting strategy that values rewards with longer delays less than hyperbolic discounting, as exponential discounting does (see Figure 1A), would maximize total gain.

The *steepness* of discounting specifies how far in the future delayed rewards should be considered. A large decay rate biases individuals to acquire small and more immediate rewards. Individuals with impulse-control disorders, as well as heroin-, alcohol-, cigarette-, and cocaine-addicted individuals, have steeper discounting functions than controls [10,26–29]. A small decay rate promotes the acquisition of large and more delayed rewards. Yet, individuals must obtain some rewards in time; for instance, an animal must find food before it starves, or before it is exhausted, or before winter arrives. Thus, the discount rate should be carefully adjusted to maximize total gain in task situations of repeated forced choices with varying delays to the rewards and limited total time [14,15].

Here, we designed a task that mimics animal foraging to study whether humans could adopt a discounting function whose shape and steepness maximize total gain. At each trial, subjects had to choose between a smaller more immediate reward (5 Japanese yen, about US$.05) and a larger delayed reward (20 Japanese yen, about US$.20), with varying experienced delays to the rewards, and fixed *ITI.* To avoid subjects trying to compute explicit reward ratios, or other objective measures of reward discounting, we did not provide direct access to the delay. Instead, subjects had to select, at each trial, between one of two squares made of 100 small patches (Figure 2). The stimulus color (white- or yellow-) coded for the monetary reward amount (5 Japanese yen and 20 Japanese yen). At each trial, the initial number of black patches in the white stimulus indicated the small delay *D_S_,* and the initial number of black patches in the yellow stimulus indicated large delay *D_L_*. The subject was then prompted to choose one of the two stimuli: the stimulus that had been selected in the previous step showed more filled patches, and the other stimulus was identical to that of the previous step. The stimuli were always displayed for one time step (1.5 s). This chain of events was repeated until either square was completely filled. Then a display of the acquired reward was shown during *ITI* = 1.5 s (see Figure 2).

**Figure 2:**
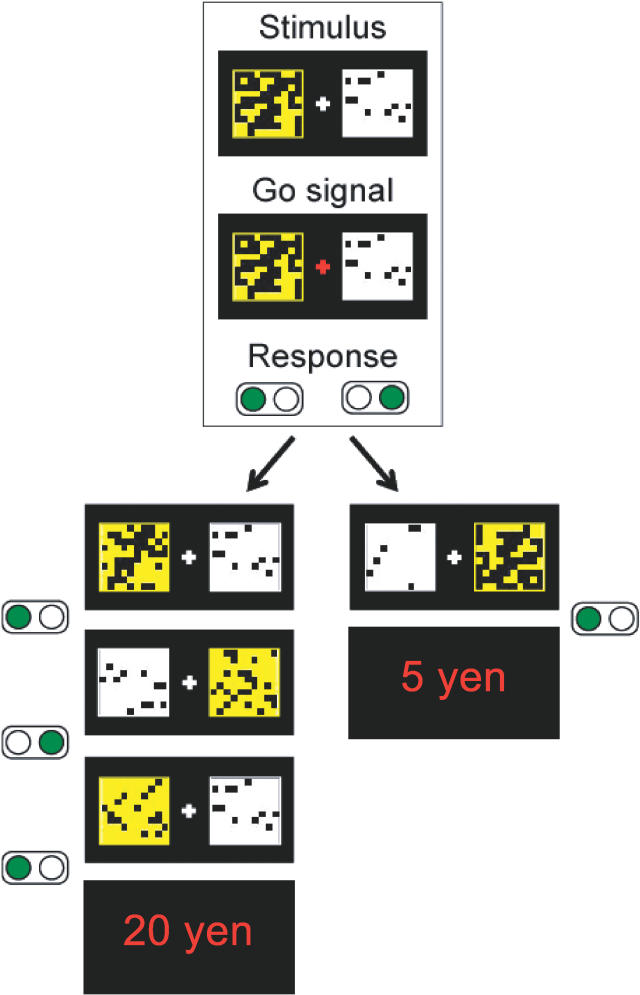
Experimental Task At each trial the subject must select either a white or a yellow mosaic after the fixation cross turns red (“Go” signal). Each button press (green disk) adds a number of colored patches to the selected mosaic. In the example shown here, if the white mosaic is selected, the subject receives 5 yen in two steps of 1.5 s each. If the yellow mosaic is selected, the subject receives 20 yen in four steps. The position of the squares (left or right) was changed randomly at each step. For each trial, the initial numbers of black patches for both mosaics were randomly drawn from uniform distributions, and indicated different delays. The *ITI,* which corresponds to the reward display, was fixed (one time step). Thus, just after the reward display, a new trial began. The subjects had a total of 700 time steps to maximize their total gain.

In the experiment, total time was limited to five sessions of 210 s each, separated by 15 s to give the subject some rest time. Thus, each subject had 700 steps (210 s * 5 sessions / 1.5 s) available to maximize the total reward. Because the subjects performed a minimum of one training session of equal duration before the experiment, they were highly familiar with the task. Subjects were compensated by the total reward earned at the end of the experiment.

## Results

With data from all trials, we first constructed *D_S_* versus *D_L_* scatter plots for each subject (Figure 3A). We first classified subjects' choices with a logistic regression model (see Materials and Methods). All models were significant (*p* < 0.05), and gave a good fit to the data: *R2_logit* = 0.55 ± 0.11 SD. An “indifference line,” for which there is equal probability to take either reward, divides the rectangular delay space into two trapezoids (see Figure 3): in the area above the indifference line, the delays *D_L_* are long, and subjects tend to select small rewards. Conversely, in the area below the indifference line, subjects tend to select large rewards. The average slope of the indifference line for all subjects was 1.1 ± 0.51 SD. Thus, on average, subjects made choices with an indifference line that is much closer to that of an exponential model—the theoretical slope is equal to 1 and independent of the rewards—than that of a hyperbolic model—the theoretical slope is equal to the ratio of the large reward to the small reward, i.e., 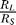 = 4 in our experiment (see Materials and Methods).

**Figure 3:**
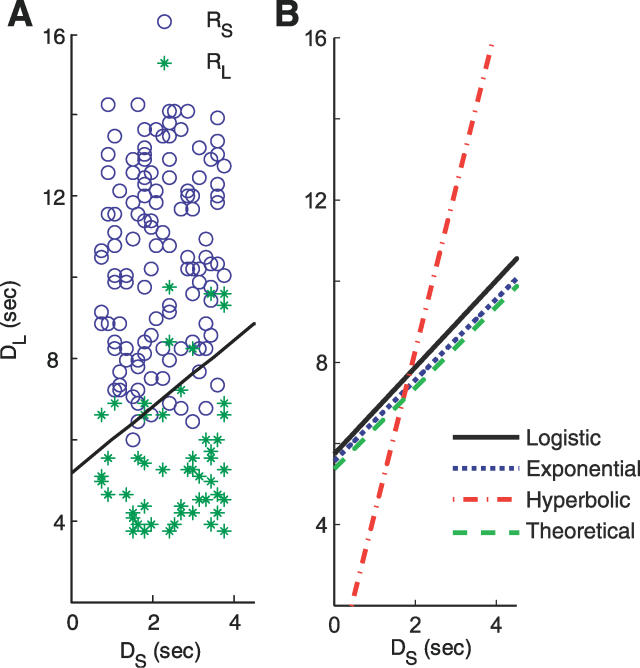
Reward Choice as a Function of Delays (A) Example of a subject's reward choice as a function of delays. At each trial, the subject had the choice between a large reward *R_L_* and a small reward *R_S_*. The indifference line (solid line) was obtained with a logistic regression model (see Materials and Methods). (B) Comparison of average indifference lines derived from the experiment with the theoretical indifference line that maximizes total gain in the experiment. Black solid line: average indifference line for all subjects obtained with the logistic regression model. Dotted blue line: average indifference line for all subjects obtained by fitting an exponential discounting model (the slope of the indifference line is 1). Dash-dotted red line: average indifference line for all subjects obtained by fitting a hyperbolic model (the slope of the indifference line is 4). Dashed green line: theoretical indifference line that maximizes the total gain in the experiment (the slope of the indifference line is 1).

Then, we directly fit the exponential model (Equation 2) and the hyperbolic model (Equations 3 and 4) to the choice data (see Materials and Methods). In each case, two parameters were estimated: *γ* and *β,* which controls the variability of reward choice, for the exponential model, *K* and *β* for the first hyperbolic model (Equation 3), and *T* and *β* for the second hyperbolic model (Equation 4). The exponential model fit gave: *γ* = 0.77 ± 0.035 SD and *β* = 7.3 ± 2.4 SD. Hyperbolic model fits gave *K* = 2.6 ± 0.94 SD and *β* = 13.8 ± 3.6 SD, and *T* = 0.30 ± 0.34 SD and *β* = 7.3 ± 3.0 SD. As can be seen in Figure 3B, the average indifference line obtained with the exponential model (i.e., with *γ* = 0.77, which corresponds to a decay rate *k* = 0.26) is close to that obtained with the logistic regression model above (compare with the line obtained with the hyperbolic model of Equation 4).

To evaluate the goodness of fit between the different two-parameter models, we computed the negative logarithm of the likelihood (*E*), also called the cross entropy error function, which is smaller for better-fitting models. Results from all subjects gave *E* = 94.6 ± 24 SD for the logistic regression model, *E* = 107 ± 25 SD for the exponential model, *E* = 161 ± 32 SD for the first hyperbolic model (Equation 3), and *E* = 155 ± 31 SD for the second hyperbolic model (Equation 4). A two-tail t-test showed that *E* for the two hyperbolic models were not significantly different (*p* = 0.47), indicating that both models fit the data equally well (this gives validity to our optimization method, as rescaling of one equation leads to the other equation). A two-tail t-test showed that *E* for the exponential model was significantly smaller than that for the hyperbolic models (*p* < 0.005 for both hyperbolic models), indicating that the exponential model better fits the data.

The generalized hyperbolic model has been proposed to be a better model of delayed reward discounting than simple hyperbolic discounting [30]. The generalized hyperbolic discounting model is given by:

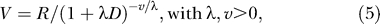
where the λ coefficient determines how much the function departs from exponential discounting. In the limit, as λ goes to zero the function becomes the exponential discounting model *V = R* exp(*−vD*). Fitting this model to the data gave: λ = 0.28 ± 0.73 SD, *v* = 0.54 ± 0.74 SD, *β* = 12.9 ± 14.2 SD, and *E* = 101 ± 23.1 SD. Despite the increase in the number of parameters from two to three, and although *E* appears to be slightly lower for the generalized hyperbolic model than for the exponential model, a two-tail t-test shows that the difference is not significant (*p* = 0.46). The slope of the indifference line for this model was 1.42 ± 0.79 SD. Interestingly, for 14 subjects, the coefficient λ was very close to zero, and the slope of the indifference line was between 1 and <1.0001, indicating pure exponential discounting for most subjects. The slope of the indifference line for four subjects was less than 2.5 (S10: 1.8, S14: 2.4, S17: 2.0, and S20 1.4), indicating near exponential discounting for these subjects. The slope for the last two subjects (S3: 3.5, and S16: 3.2) was close to the ratio of the large to the small reward, that is, 4, indicating discounting closer to hyperbolic discounting for these subjects.

Next, we estimated the coefficients of a semiparametric value model with exponential basis functions (see Materials and Methods). Because integrating the exponential discounting function with respect to the decay rate *k* from 0 to infinity yields a hyperbolic function of the delay *D,* a sum of several exponentials with different decay rates approximates a hyperbola [31]. Thus, if a number of coefficients in the semiparametric model are positive, subjects would discount reward approximately hyperbolically. In contrast, if only one or a few nearby coefficients are positive, then subjects would discount reward exponentially. Figure 4 shows that the distribution of coefficients was sparse: all subjects exhibited a single narrow first peak (peak width: 0.050 ± 0.008 SD sec^−1^). Further, the average decay rate was 0.25 ± 0.06 SD sec^−1^ (a very similar average decay rate was obtained with the direct exponential fit method—see above), with a sharp distribution ranging between 0.13 and 0.35 sec^−1^. For 13 subjects, this peak was the only peak, indicating pure exponential discounting. For seven subjects the first peak was followed by a prominent second peak; two of these subjects had a secondary isolated peak (near *k* = 0.75 sec^−1^), and for five of these subjects, a higher frequency component appeared at *k* = 1 sec^−1^ (e.g., subject 3). This method confirmed the results of the generalized hyperbolic model fit, as 13 subjects were identified as pure exponential discounters by both methods.

**Figure 4:**
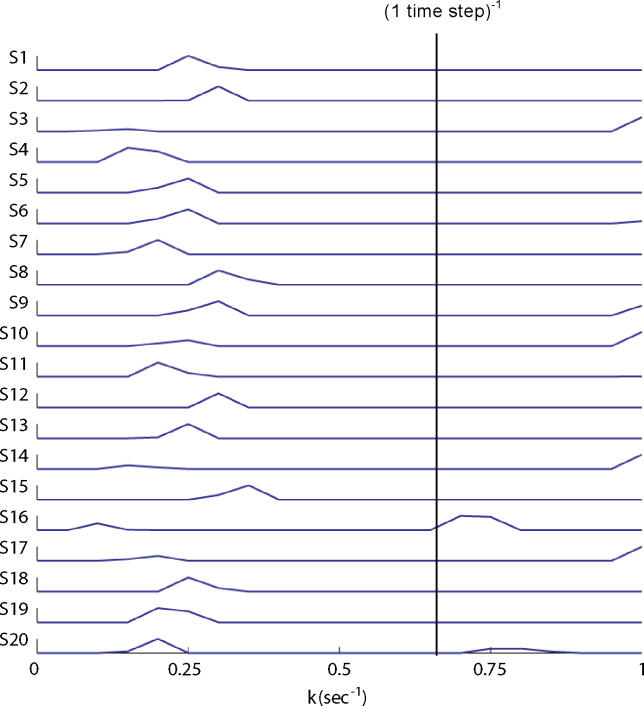
Coefficients of the Exponential Basis Functions Normalized to Unity for Each of the 20 Subjects (S1 to S20) Note the sparseness of the coefficient distribution: all subjects exhibit a single peak for decay rates in the range 0.125 and 0.35 sec^−1^.

In our experiment, the subjects gained an average of 1840 ± 71 SD yen. Could the subjects have earned more if they had adopted different decision lines? In other words, were the subjects' choices optimal with regard to maximizing their total gain? To answer this question, we estimated the indifference line that yields the maximum theoretical total reward in our experimental setting, independently of any particular model (hyperbolic or exponential). We first computed the expected reward rate—the “value” *V* (see Materials and Methods). Then, we computed the maximum expected reward rate *Vmax,* by computing the two partial derivatives of the expected reward rate with respect to the slope *a* and the intercept *b* of the indifference line *D_L_* = *aD_s_* + *b*. A maximum of *V* is obtained when both partial derivatives are equal to zero.

We found only one (real number) solution with respect to the slope *a* of the indifference line*, a* = 1, and one (real number) solution for the intercept that maximizes *V, b* = 6.93. Furthermore, taking into account the *ITI,* the intercept corresponds to an exponential decay rate of *k* = 0.25 (discount rate 0.77), very close to the average decay rate of our subjects (average decay rate found with the exponential model: *k* = 0.26). Thus, our analytical analysis shows that the theoretical indifference line is very close to the lines obtained with the logistic regression model fit and with the exponential model fit (see Figure 3B). It is also noteworthy that the slope *a* = 1 that maximizes *V* was independent of the maximum and minimum of the boundaries of the (*D_S_, D_L_*) space *(α, β, η,* and *μ*), and independent of the *ITI* as well.

Finally, using an optimization method (see Materials and Methods), we then confirmed that we did not find such an indifference line “by chance”: any experiment similar to ours, but with different boundaries of the (*D_S_, D_L_*) space, different rewards, and/or different *ITI,* would also yield an indifference line of slope 1. Table 1 shows that the optimization method gives the same results as the exact analytical method for the experimental parameters (“original parameters”). Further, although the intersect value *b* and the maximum reward rate *Vmax* depended on the various experimental parameters, the slope *a* stayed exactly equal to 1 as we varied the experimental parameters.

**Table 1:**
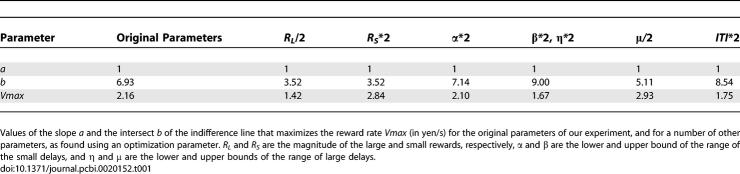
Parameter Sensitivity Analysis

## Discussion

In our experiment, most of our subjects adopted a discounting function with a *shape* (exponential) and *steepness* (the decay rate) appropriate to maximize the total reward in the experiment. Using a logistic regression model, we found that the average indifference line had a slope near 1, as predicted by exponential discounting. Then, a direct fit of the data with exponential and hyperbolic models indicated that the exponential model better fitted the data overall. A fit with the generalized hyperbolic model [30] showed pure exponential discounting for 14 out of 20 subjects, and near exponential discounting for three more subjects. Next, using a semiparametric method to approximate the value function with exponential bases, we found a sparse distribution of positive basis coefficients, with a single isolated peak for most subjects, further supporting exponential discounting. Finally, we showed both analytically and with an optimization technique that the theoretical indifference line that maximizes the total gain in our experiment had a slope of exactly 1. Importantly, this result was not affected by the magnitude of the reward ratio; thus, we predict that this result would hold for different reward magnitudes. However, as it has been suggested that the value of a positive reinforcer increases as a hyperbolic function of its size [11], this prediction needs to be further tested.

The use of exponential discounting by our subjects appears to be a farsighted strategy that allows an optimal tradeoff between (the relatively short) delays at each trial and (the relatively long) total time remaining in the experiment. The use of hyperbolic discounting, in contrast, would be a greedy, but myopic strategy, which would maximize the instantaneous reward rate at each trial, not the total reward gain. Thus, our results suggest that humans can overcome their hyperbolic discounting when it is suboptimal, and discount time exponentially instead to maximize total gain.

Not all our subjects exhibited pure exponential discounting, however. Our direct-fit method using the generalized hyperbolic model notably showed that two subjects exhibited discounting closer to hyperbolic discounting, and four subjects exhibited intermediate discounting closer to exponential discounting. Our semiparametric method mostly yielded similar results, with the addition of one other near-exponential discounter. These subjects had a discount function with two decay rates: one similar to the other subjects, around 0.25 sec^−1^, and a second higher decay rate above 0.67 sec^−1^. Because the time step in the experiment was 1.5 s, we can interpret any decay rate beyond 0.67 sec^−1^ as the bias for a small reward choice available within one step (see Figure 4). Thus, for these subjects, the discounting functions are qualitatively similar to that proposed by the quasi-hyperbolic model [32,33], for which initial discounting after the first time step is steeper than subsequent discounting, which is exponential.

The decay rates used by our subjects were in good agreement with the theoretical discount rate that maximizes the total gain. These decay rates were close to that observed in animal studies [34], and similar to that reported in a related human experiment [20], but several order of magnitudes larger than that observed in other questionnaire-based human studies [19], suggesting that humans can select decay rates based on the task at hand. Note that our optimization methods give us an overall estimate of the discount factor, that is, it does not allow us to tract variations, if any, of the discount factor within the session. However, since the subjects had one training session before the experiment, it is probable that most meta-learning of the discount parameter occurred previous to the experiment.

Although, to our knowledge, exponential discounting had not been previously demonstrated in human reward discounting, a number of investigators have suggested that, in some circumstances, humans can be less impulsive than predicted by hyperbolic discounting, and behave in a more rational manner. Forzano and Logue [35] showed that subjects are more impulsive in conditions when juice is given during the experiment (after each choice), compared with conditions when subjects are given money or points exchangeable for a total (juice) reward at the end of the experiment (as in the present experiment). Loewenstein [36] pointed out that people are impulsive as a result of the effect of visceral factors, such as hunger, thirst, and sexual desire, on the desirability of immediate consumption. When no immediate visceral factors are involved, people tend to be less impulsive. Montague and Berns [25] proposed that because of uncertainty in reward estimation, reward values should be more steeply discounted than exponential discounting. However, according to their model, if there is no uncertainty of reward estimation, then discounting is exponential. Finally, Read [37] showed that humans do not discount rewards hyperbolically but subadditively, that is, they tend to discount rewards more if the delay is divided into subintervals than when it is left undivided. Subadditivity is then explained by a modified exponential function, where the delay *D* is taken to the power of a parameter *s,* 0 < *s* < 1 reflecting nonlinear time perception. As this parameter approaches 1, discounting becomes exponential.

What may be the possible neural correlates of exponential or hyperbolic discounting? We have previously found that parallel cortico–basal ganglia loops are involved in reward prediction with different discounting factors [38]. Because summation of several exponential discounting can yield hyperbolic discounting [31], simultaneous activation of a number of exponential parallel cortico–basal ganglia loops could generate hyperbolic discounting. If reward prediction at a larger time scale is required, as in questionnaire-based human experiments, the frontal cortex would be additionally recruited [39]. If, however, exponential discounting of rewards at relatively short delays is required, as in the present experiment, a particular cortico–striatial loop with the appropriate discount rate would be selected, possibly via serotonin modulation (Tanaka SC, Schweighofer N, Asahi S, Okamoto Y, Yamawaki S, et al. (2006) Serotonin regulates striatal activities in delay discounting, unpublished data).

## Materials and Methods

### Subjects.

Twenty-two healthy, right-handed male volunteers, with no history of psychiatric or neurological disorders, gave written informed consent after the nature and possible consequences of the study were explained. The study was approved by the ethics and safety committees of the Advanced Telecommunications Research Institute International and of Hiroshima University. We recruited only male subjects to avoid estrogen-level fluctuation during the menstrual cycle in women, which affects central serotonin levels. The results reported here are part of an experiment to study the role of serotonin in reward choice and learning. In this within-subject experiment, six hours before the beginning of the behavioral task, the subject consumed one of three amino acid drinks: one containing a standard amount of tryptophan (2.3 g per 100 g amino acid mixture), one containing excess tryptophan (10.3 g), and one without tryptophan (0g )—more experimental details of serotonergic manipulation are described elsewhere (Tanaka SC, Schweighofer N, Asahi S, Okamoto Y, Yamawaki S, et al. (2006) Serotonin regulates striatal activities in delay discounting, unpublished data). Here, we present the results for twenty subjects in the control condition, who drank the solution containing the standard amount of tryptophan. The mean plasma-free tryptophan concentrations at the time of the experiment in the control condition was 2.42 ± 0.98 SD mg/ml. These levels are slightly higher than normal physiological levels, about 1.3–1.5 mg/ml [40–42], but much lower than those in the high-tryptophan condition (61.2 ± 34 SD mg/ml).

Two subjects were excluded from the study. The first subject was excluded because no change in plasma-free tryptophan measurements between the control-tryptophan and the high-tryptophan conditions could be detected. This can be explained by either an error in the procedure, or by digestive problems, as all other subjects exhibited a dramatic increase in plasma-free tryptophan measurements in the high-tryptophan condition (close to a 40-fold increase compared with preingestion measurements). The second subject was excluded because of a technical problem that prevented us from recording the choice data in the low-tryptophan condition.

### Task.

Two stimuli (one white-coded for the small reward, and one yellow-coded for the large reward) were presented during a time selected from a uniform distribution ranging from 0.4 to 0.7 s from the onset of the presentation of the stimuli. Then, a change of color in the fixation cross from white to red acted as a “Go” signal; then the subject had to decide to pursue either the large or the small reward. The subject then clicked on the mouse button associated with the position of the chosen stimulus (i.e., left button to choose the left stimulus, for instance). After 1.5 s from the beginning of the step, two new stimuli were presented, and a new step started—the stimulus that was chosen showed more filled patches and the stimulus that was not chosen was identical to that of the previous step. A trial ended when either square was completely filled (100 patches were filled). The corresponding monetary reward then appeared on the screen for 1.5 sec (corresponding to an *ITI* of 1.5 s). To maintain the subjects' attention, the position of the squares (left or right) was changed randomly at each step.

At each trial, the delays to the small and large rewards *D_S_* and *D_L_* are theoretically given by:

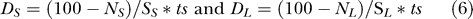
where *ts* is the time step (1.5 s), *N_S_* and *N_L_* are the initial number of white and yellow patches, and *S_S_* and *S_L_* are the number of patches added per step (10 ± 2 patches/step). At the onset of each trial, the white and yellow patches were drawn from random uniform distributions: white patches were in the range 85 ± 10 and initial yellow patches in the range 40 ± 35. Thus, the white square always appeared brighter than the yellow square on the first step of each trial, and the average delay needed to get a large reward was 4× that to get a small reward (excluding the *ITI*). For the average value of *S_S_* and *S_L_,* the range of theoretical delays for the small rewards was 0.75 to 3.75 s, and for the large rewards 3.75 to 14.25 s. Because the experimental step was 1.5 s, however, the actual delays were the delays above rounded to the next 1.5-s increment; further, every trial also contained an additional step due to *ITI* = 1.5 s.

### Data analysis.

We first approximated the choices with a two-parameter logistic regression model:

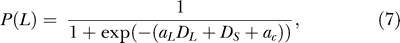
where *P*(*L*) is the probability to choose the large reward, *a_L_,* and *a_c_* are parameters that were determined using the Matllab function *glmfit* with a maximum likelihood loss function. Note that for the logistic regression model of Equation 7, −1*/a_L_* gives the slope of the indifference line (for which *P*(*L*) = 0.5).

Then, we directly fit different discounting models to the data. For this, we used the following equation, which gives the probability of choosing the large reward:

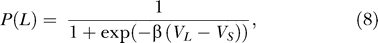
where *V_L_* and *V_S_,* are the large and small reward values, and *β* the “inverse temperature,” which controls the randomness of the reward choice. *V* was replaced by Equation 2 (exponential model), Equations 3 and 4 (hyperbolic models), and Equation 5 (generalized hyperbolic model).

By taking *V_L_* = *V_S_,* it can be easily shown that the indifference line's equation for the exponential discounting model is:

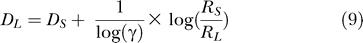



The slope of the indifference of line is 1, independent of the reward amounts. For the hyperbolic model, the indifference line is:

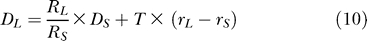
The slope of this line is the ratio of the rewards 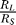 ; thus, in our specific case, the slope is 40/10 = 4 (Note: for the other form of the hyperbolic model, it can easily be shown that the slope is also the ratio of the rewards). For the generalized hyperbolic model, the indifference line is given by:

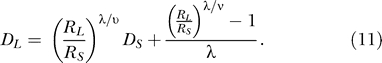



The parameters for these three models (*k* and *β* for the exponential model, *K,* or *T* and *β* for the hyperbolic models, and *λ, v,* and *β* for the generalized hyperbolic model) were constrained the be positive (≥0), and were found by fitting the models to the subjects' choices with a maximum likelihood loss function. Such optimization can be performed using sequential dynamic programming, which is available in Matlab using the optimization function *fmincon*. This function estimates the Hessian of the Lagrangian through the BFGS formula at each iteration. Then, the line search method is used with this estimation to find the parameters that minimize the maximum likelihood loss function.

Next, we estimated the discounting function directly with a semiparametric model. Specifically, each value function was computed as a weighted sum of exponential basis functions:

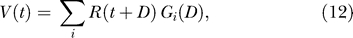
where the basis functions were given by

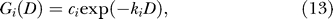
Where 0 ≤ *k* ≤ *k*_max_ and *c_i_* are the basis coefficients. We replaced the two value functions in Equation 8 by their semiparametric expression, which gives:

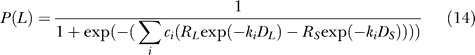



The basis coefficients *c_i_* were constrained to be positive, and were found by fitting subjects' choices with a maximum likelihood loss function. The optimization was performed with the function *fmincon,* as above. We estimated the coefficients for decay rates *k* between 0 and 1 sec^−1^ with increments of 0.05 sec^−1^.

### Mathematical analysis.

To estimate the indifference line that gives the maximum theoretical total reward, we computed the expected reward rate (in yen/s), given by:

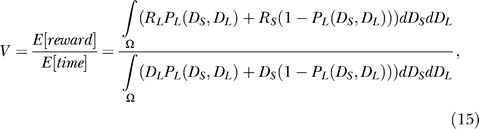
where Ω is the *D_L_*,*D_S_* space, modified by rounding the space boundary to the next time step and by adding the *ITI,* and *P_L_*(*D_S_, D_L_*) is the probability of choosing the large reward for the delays *D_S_* and *D_L_*. The total reward is then the reward rate times the total time in the experiment. We parameterized the expected reward rate with a family of indifference line modeled with

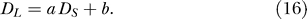



To find the parameters *a* and *b* that maximize the value given by Equation 15, we simplified the problem by assuming that subjects made deterministic decisions. If in a one-trial, *D_L_* ≤ *aD_S_* + *b,* then the large reward is chosen; the small reward is chosen otherwise. We then noted that the value function equation can be evaluated with two separated trapezoids, one above and the other below the indifference line. Thus, *E[reward]* consists of two terms.

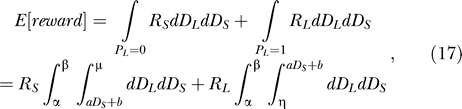
where *α*, *β*, *η*, and *μ* are the lower and upper bounds of the *D_L_*,*D_S_* space. Similarly

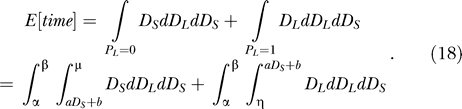



We then computed the partial derivative of *V* with respect to the parameters,*a* and *b:*

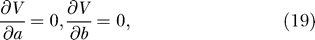
and solved these equations analytically using Mathematica software.

### Sensitivity analysis.

We used an optimization method (1) to verify our analytical results and (2) to perform a sensitivity analysis to examine how variations in experimental parameters affected the values of *a* and *b* that maximized the expected reward rate *V*. We computed the maximum of *V* using the Matlab function *fminunc,* which is similar to the function *fmincon,* but without any constraints on the parameters.

## Abbreviation

*ITI***: inter-trial interval
